# Use of T Cell Mediated Immune Functional Assays for Adjustment of Immunosuppressive or Anti-infective Agents in Solid Organ Transplant Recipients: A Systematic Review

**DOI:** 10.3389/fimmu.2020.567715

**Published:** 2020-10-15

**Authors:** Omid Rezahosseini, Dina Leth Møller, Andreas Dehlbæk Knudsen, Søren Schwartz Sørensen, Michael Perch, Finn Gustafsson, Allan Rasmussen, Sisse Rye Ostrowski, Susanne Dam Nielsen

**Affiliations:** ^1^Viro-Immunology Research Unit, Department of Infectious Diseases 8632, Rigshospitalet, University of Copenhagen, Copenhagen, Denmark; ^2^Department of Cardiology, Rigshospitalet, University of Copenhagen, Copenhagen, Denmark; ^3^Department of Nephrology, Rigshospitalet, University of Copenhagen, Copenhagen, Denmark; ^4^Department of Clinical Medicine, University of Copenhagen, Copenhagen, Denmark; ^5^Department of Cardiology, Section for Lung Transplantation, Rigshospitalet, University of Copenhagen, Copenhagen, Denmark; ^6^Department of Surgical Gastroenterology and Transplantation, Rigshospitalet, University of Copenhagen, Copenhagen, Denmark; ^7^Department of Clinical Immunology, Rigshospitalet, University of Copenhagen, Copenhagen, Denmark

**Keywords:** transplantation, immune system, immunosuppressive agent, anti-infective agent, immune functional assay

## Abstract

**Background:** Defining the optimal dosage of the immunosuppressive or duration of anti-infective agents is a challenge in solid organ transplant (SOT) recipients. We aimed to systematically review the literature regarding the use of T cell mediated immune functional assays (IFAs) for adjustment of the immunosuppressive or anti-infective agents in SOT recipients.

**Methods:** We systematically searched PubMed, Scopus, EMBASE, Web of Science (WOS), Cochrane Central Register of Controlled Trials (CENTRAL), and ClinicalTrials.gov to find human interventional studies or study protocols that used either in-house or commercially available IFAs for adjustment of the immunosuppressive or anti-infective agents in SOT recipients.

**Results:** We included six clinical trials and six study protocols. Four out of the six clinical trials used interferon-γ release assays for cytomegalovirus (IGRA-CMV), and five out of the six registered study protocols planned to use IGRA-CMV for adjustment of anti-CMV antiviral (Valganciclovir) prophylaxis or preemptive therapy in SOT recipients. Primary or secondary anti-CMV prophylaxes were discontinued in SOT recipients who had positive IGRA-CMV results without an increase in the rate of CMV infection or reactivation. Among other IFAs, one clinical trial used interferon-γ release assays for tuberculosis (IGRA-TB), and one study used ImmuKnow for adjustment of the duration and dosage of isoniazid and tacrolimus, respectively.

**Conclusion:** Our systematic review supports a promising role for the IGRA-CMVs for adjustment of the duration of anti-CMV antiviral prophylaxis in SOT recipients. There are limited data to support the use of IFAs other than IGRA-CMVs for adjustment of immunosuppressive or anti-infective agents. Further multicenter randomized clinical trials using IFAs other than IGRA-CMVs may help in personalized immunosuppressive or prophylactic anti-infective therapy in SOT recipients.

## Introduction

Solid-organ transplantation (SOT) is a life-saving treatment option for patients with terminal organ failure (1). To avoid rejection of the transplanted organ, SOT recipients receive life-long immunosuppressive therapy (1). Immunosuppressive therapy, however, is a double-edged sword; over-immunosuppression may precipitate cancers and infections, while under-immunosuppression increases the risk of graft rejection (2, 3). Consequently, monitoring of the immunosuppressive drug-level is part of the standard of care in SOT recipients. However, infection rates may differ in SOT recipients who receive the same immunosuppression regimen, and with equal trough level of the immunosuppressive agents (4). Moreover, SOT recipients usually receive a combination of immunosuppressive agents with different mechanisms of action (5, 6), and therapeutic drug monitoring of individual drugs may not accurately reflect the immune status. To prevent infections, SOT recipients may need prophylactic anti-infective agents for cytomegalovirus (CMV) disease, tuberculosis, pneumocystis pneumonia (PCP), and some other infective agents but there is no accurate measure to guide optimal duration of the prophylactic anti-infective agents in SOT recipients (7, 8). Thus, more precise tools to monitor the function of the immune system in SOT recipients and to guide the dosing of immunosuppressants and anti-infective agents are needed.

Immune functional assays (IFAs) are assays that use a stimulant to trigger the immune cells and afterward record the functional immune response (4, 9–11) As such, IFAs may be used to monitor the immune function and could be used in approaches toward personalized treatment with immunosuppressive and anti-infective agents. Several standardized (commercially available) and in-house IFAs have been introduced. A common feature of these IFAs is that mainly test T cell function (11–14). We aimed to systematically review the literature regarding the use of T cell mediated IFAs for adjustment of the immunosuppressive or anti-infective agents in SOT recipients.

### A Summary of the Available IFAs

Several commercially available *in vitro* IFAs are routinely used in research and clinical practice (9, 10). Assays such as ImmunKnow®, QuantiFERON®-TB Gold, T-SPOT®.TB, QuantiFERON®-CMV, T-Track® CMV, and T-SPOT®.CMV, are examples of commercially available IFAs (9–11). Below we summarize the mechanism of action of the mentioned commercially available *in vitro* IFAs used in solid organ transplantation.

#### ImmuKnow®

**Figure 1:**
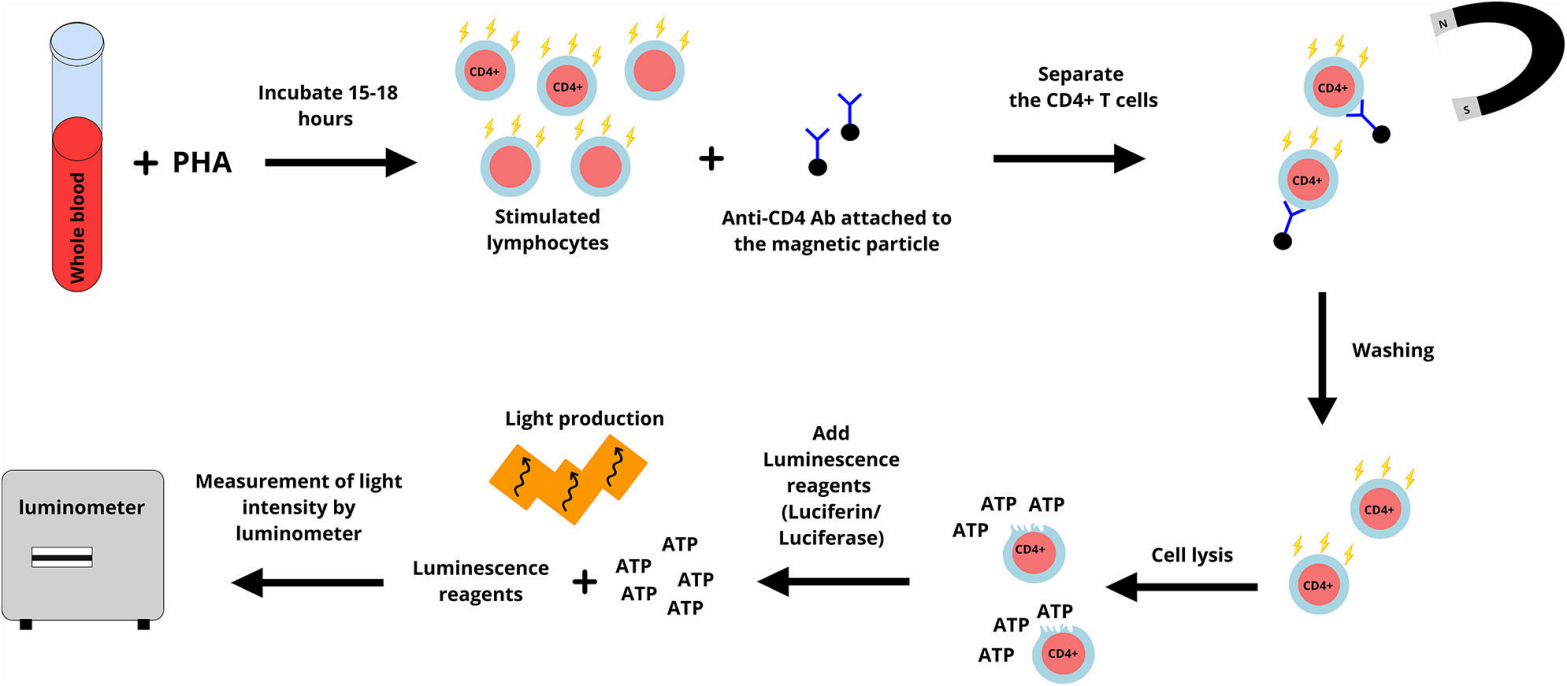
In ImmuKnow assay, a sample of whole blood is incubated with phytohemagglutinin (PHA) for 15–18 h. PHA stimulates lymphocytes and lymphocytes including CD4+ T lymphocytes produce adenosine triphosphate (ATP). Anti-CD4 antibodies attached to magnetic particles are added and attach to CD4+ T lymphocytes. CD4+ T lymphocytes are purified and after washing, are lysed. A luminescence reagent (a mixture of luciferin and luciferase) is added. ATP activates luciferase and this enzyme cut luciferin which results in light production. The produced light is measured using the bioluminescence method and reported in nanograms per milliliter (ng/ml). The figure designed by authors using Gravit Designer.

Most of the cell functions are dependent on the production of adenosine triphosphate (ATP) and intracellular synthesis of ATP is a marker of cell activity (15). ImmunKnow® (Cylex, Columbia, USA) uses this principle to measure the activity of CD4+ T lymphocytes (10). In ImmuneKnow®, phytohemagglutinin (PHA) is used to stimulate lymphocytes, and the concentration of ATP is measured using the bioluminescence method (Figure 1) and reported in nanograms per milliliter (ng/ml) (10). According to the recommendations by manufacturer, ATP levels equal to or lower than 225 ng/mL are interpreted as a low immune cell response, while ATP levels equal to or higher than 525 ng/mL are interpreted as a high immune cell response (10).

T-lymphocytes are the main target of most immunosuppressive agents, including cyclosporine and tacrolimus (5). Therefore, the ImmunKnow® assay is relevant as an IFA to monitor the immune system in SOT recipients. Immunosuppressive agents are present both in plasma and inside the red blood cells, ImmunKnow® is performed on a sample of whole blood to ensure that lymphocytes are tested in the presence of immunosuppressive drugs. Moreover, pre-purification of lymphocytes is not necessary to perform the ImmunKnow® assay and this decreases the iatrogenic stimulation of lymphocytes (10). Using whole blood is one of the advantages of this assay (16). However, PHA is not a specific stimulator of CD4+ T lymphocytes, and all types of living cells produce ATP. Furthermore, the requirement for purification and lysis of CD4+ T lymphocytes after stimulation are among the disadvantages of ImmunKnow®. This assay was approved by U.S. Food and Drug Administration (FDA) (17).

#### Interferon-Gamma Release Assays for Tuberculosis (IGRA-TB)

##### QuantiFERON^®^-TB Gold and T-SPOT^®^.TB assays

**Figure 2:**
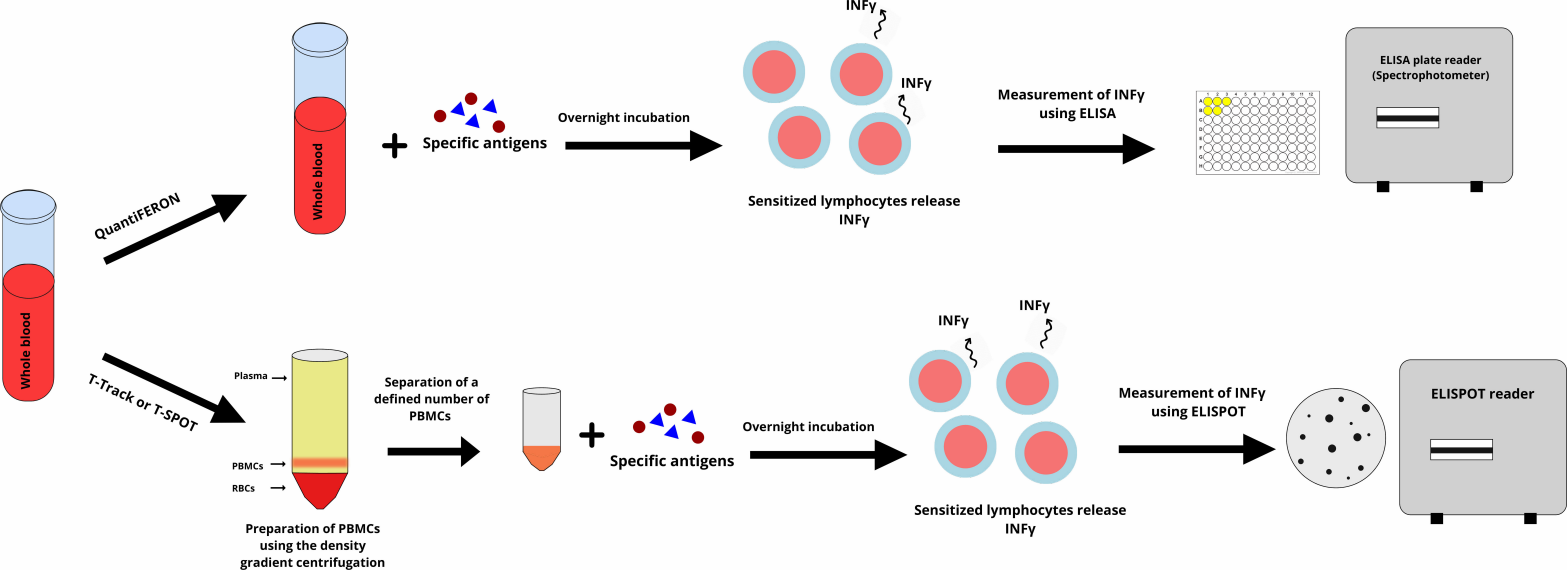
The Interferon-Gamma Release Assays (IGRAs) is performed according to two different methods. In the QuantiFERON, whole blood is incubated with specific antigens overnight (16–24 h). Antigens stimulate lymphocytes and sensitized lymphocytes release interferon-gamma. The interferon-gamma is measured using an enzyme linked immunosorbent assay (ELISA) and is reported as international units (IU) per milliliter. In the T-Track or T.SPOT assay peripheral blood mononuclear cell (PBMC) purified from whole blood are incubated with specific antigens overnight, and sensitized PBMCs release interferon-gamma. The interferon-gamma is measured using an enzyme-linked immunosorbent spot (ELISPOT) and is reported as the number of the formed spots on the ELISPOT's plate. The figure designed by authors using Gravit Designer.

The interferon-gamma release assay for tuberculosis (IGRA-TB) is an *in vitro* assay that measures the production of IFN-γ following stimulation of T lymphocytes with *Mycobacterium tuberculosis* (*M*. *tuberculosis*) specific antigens (Figure 2). The antigens used are highly specific for *M*. *tuberculosis* and are not found in most of the non-tuberculous mycobacterium, including Bacillus Calmette-Guérin (BCG) (18). Currently, there are two commercially available IGRA-TB, the QuantiFERON®-TB Gold (Qiagen, Hilden, Germany) and T-SPOT®.TB assay (Oxford Immunotec, Abingdon, UK) (11, 18–20).

In the QuantiFERON®-TB Gold In-Tube, whole blood is incubated with the *M. tuberculosis* specific antigens (early secreted antigenic target 6 (ESAT-6), culture filtrate protein 10 (CFP-10), and TB 7.7. The IFN-γ in the supernatant is measured using an enzyme-linked immunosorbent assay (ELISA) technique (Figure 2) and reported as international units (IU) per milliliter (18, 21). QuantiFERON®-TB Gold Plus is a new generation of QuantiFERON®-TB Gold that uses a peptide cocktail that mimick the ESAT-6 and CFP-10 antigens and stimulate CD4+ T lymphocytes, plus an additional set of peptides that stimulate CD8+ T lymphocytes (19).

In T-SPOT®.TB assay, peripheral blood mononuclear cells (PBMCs) are separated from a sample of whole blood using the Ficoll method (density gradient centrifugation), and a defined number of PBMCs are incubated with *ESAT-6 and CFP 10* resulting in release of IFN-γ from sensitized T lymphocytes (20, 22). T-SPOT®.TB is an enzyme-linked immunosorbent spot (ELISPOT) assay with pre-coated plates with anti-IFN-γ antibodies (Figure 2) (11, 18). The use of highly M. tuberculosis specific antigens is one of the advantages of the QuantiFERON®-TB Gold and T-SPOT®.TB assays. This decreases the risk of false-positive results in individuals who have received the BCG vaccine or who are infected with other strains of mycobacteria. IGRAs, however, have a high number of indeterminate results and poor reproducibility mainly in immunocompromised patients (23–26). In comparison with QuantiFERON®-TB Gold, the T-SPOT®.TB assay is more laborious, takes more time, and needs specific laboratory equipment for purification of PBMCs (27, 28). Both assays approved by FDA and QuantiFERON®-TB Gold has the Conformitè Europëenne Mark (CE Mark) for commercial use in Europe (19, 20).

#### Interferon-Gamma Release Assays for Cytomegalovirus (IGRA-CMV)

##### QuantiFERON^®^-CMV and T-Track^®^ CMV

The principal of the IGRA-CMV is similar to the IGRA-TB (Figure 2), however, CMV specific antigens are used in IGRA-CMV (29). Three commercially available IGRA-CMV are available. QuantiFERON®-CMV (Qiagen, Hilden, Germany), T-Track® CMV assay (Lophius, Regensburg, Germany), and T-SPOT®.CMV (Oxford Immunotec, Abingdon, UK) (29–31).

The QuantiFERON®-CMV assay monitors the immune response to the cytomegalovirus (CMV). QuantiFERON®-CMV uses human leukocyte antigen (HLA) class I specific synthetic CMV epitopes to stimulate CMV-specific CD8+ T lymphocytes. These epitopes are peptide sequences of pp65, pp50, immediate-early 1 (IE-1), and the glycoprotein gB antigens (Figure 2) (29).

T-Track® CMV assay stimulates PBMCs with recombinant pp65 and IE-1 antigens that are called T-activated® proteins. These T-activated® proteins have been processed and can stimulate CD4+ T lymphocytes, CD8+ T lymphocytes, and natural killer cells (Figure 2) (30). In terms of principal, T-SPOT®.CMV is similar to T-Track CMV and uses pp65 and IE-1 CMV antigens as the stimulator (31).

When comparing QuantiFERON®-CMV, T-Track® CMV, and T-SPOT®.CMV, the QuantiFERON®-CMV is easier to perform as it does not need laboratory instruments for purification of PBMCs (Figure 2) (31). However, the ELISPOT based assays are more sensitive than ELISA based assays for the detection of cytokines including IFN-γ in the supernatant (32). QuantiFERON®-CMV, T-Track® CMV, and T-SPOT®.CMV are not FDA approved, however are CE Marked for commercial use in Europe (31, 33, 34).

## Materials and Methods

### Search Strategy

We used the preferred reporting items for systematic reviews and meta-analyses (PRISMA) statement (35). The clinical question was designed according to the PICOS process and keywords selected to cover the clinical question (36). We searched PubMed, Scopus, EMBASE, Web of Science (WOS), Cochrane Central Register of Controlled Trials (CENTRAL), and ClinicalTrials.gov from 1 January 1970 to 15 May 2020. In a complementary search, we did a manual search in google using the mentioned keyword combinations. We also studied the reference list of eligible studies for any additional relevant papers.

Two independent investigators searched the mentioned databases using the same search terms and screened the retrieved papers by title and abstract. Potentially relevant papers were read in full-text and included if inclusion criteria were fulfilled.

The study protocol of this systematic review was registered on the international prospective register of systematic reviews (PROSPERO) with registration number of CRD42020182068.

### Inclusion Criteria

We included studies on human recipients of solid organs of any type and any age group. The studies should use at least one of the commercially available or in-house IFAs for adjustment of dosage or duration of immunosuppressive and/or prophylactic anti-infective agents. We included interventional studies including randomized or non-randomized clinical trials. In regard to the type of articles only original articles, brief reports, research correspondence, or study protocols in English were included. We excluded studies that did not full-fill the inclusion criteria. We also excluded review articles, case reports, and conference proceedings.

### Full Electronic Search Strategy in PubMed

We used the following MeSH terms in PubMed from 1 January 1970 to 15 May 2020 and found 75 hits.

(((((((“Interferon-gamma Release Tests” [Mesh]) OR “Enzyme-Linked Immunospot Assay” [Mesh]) OR “Phytohemagglutinins” [Mesh]) OR “Lipopolysaccharides” [Mesh]) OR “Mitogens” [Mesh])) AND (((“Antibiotic Prophylaxis” [Mesh]) OR “Immunosuppressive Agents” [Mesh]) OR “Anti-Infective Agents” [Mesh])) AND “Organ Transplantation”[Mesh].

Using the free-text terms in the same time-period, we found 797 hits in PubMed.

(((((((((((IGRA) OR QuantiFERON) OR ImmuKnow) OR T-Track) OR T.SPOT) OR TruCulture)) OR ((ELISpot) OR FluoroSpot))) OR ((((phytohaemagglutinin) OR lipopolysaccharides) OR proliferation assay) OR in-house assay))) AND ((((prophylaxis^*^) OR antibiotic) OR antiviral) OR immunosuppress^*^)) AND organ transplant^*^.

We used the same combination of the free-text terms to search Scopus, EMBASE, WOS, CENTRAL, and CinicalTrials.gov.

### Data Extraction and Risk of Bias of the Included Studies

The selected papers were studied in full text by the same two investigators who did the search process (OR and DLM). We used the Cochrane risk of bias tools for randomized (RoB 2.0) and non-randomized (ROBINS-I) clinical trials (37, 38). We also used the online version of *robvis* (visualization tool) to draw the traffic light plots and weighted bar plots. Risk of bias tools use standard signaling questions and elicit aspects of a clinical trial that are related to the risk of bias. According to an algorithm and based on the answers to the signaling question, the risk of bias is evaluated (37, 38). Using traffic light plots and weighted bar plots, the judgment for risk of bias in randomized clinical trials is shown as either low, some concerns, or high, and the judgment for non-randomized clinical trials is shown as low, moderate, or high (37, 38).

The included studies were not homogenous, hence we could not perform a meta-analysis and only used narrative data synthesis for the results.

## Results

**Figure 3:**
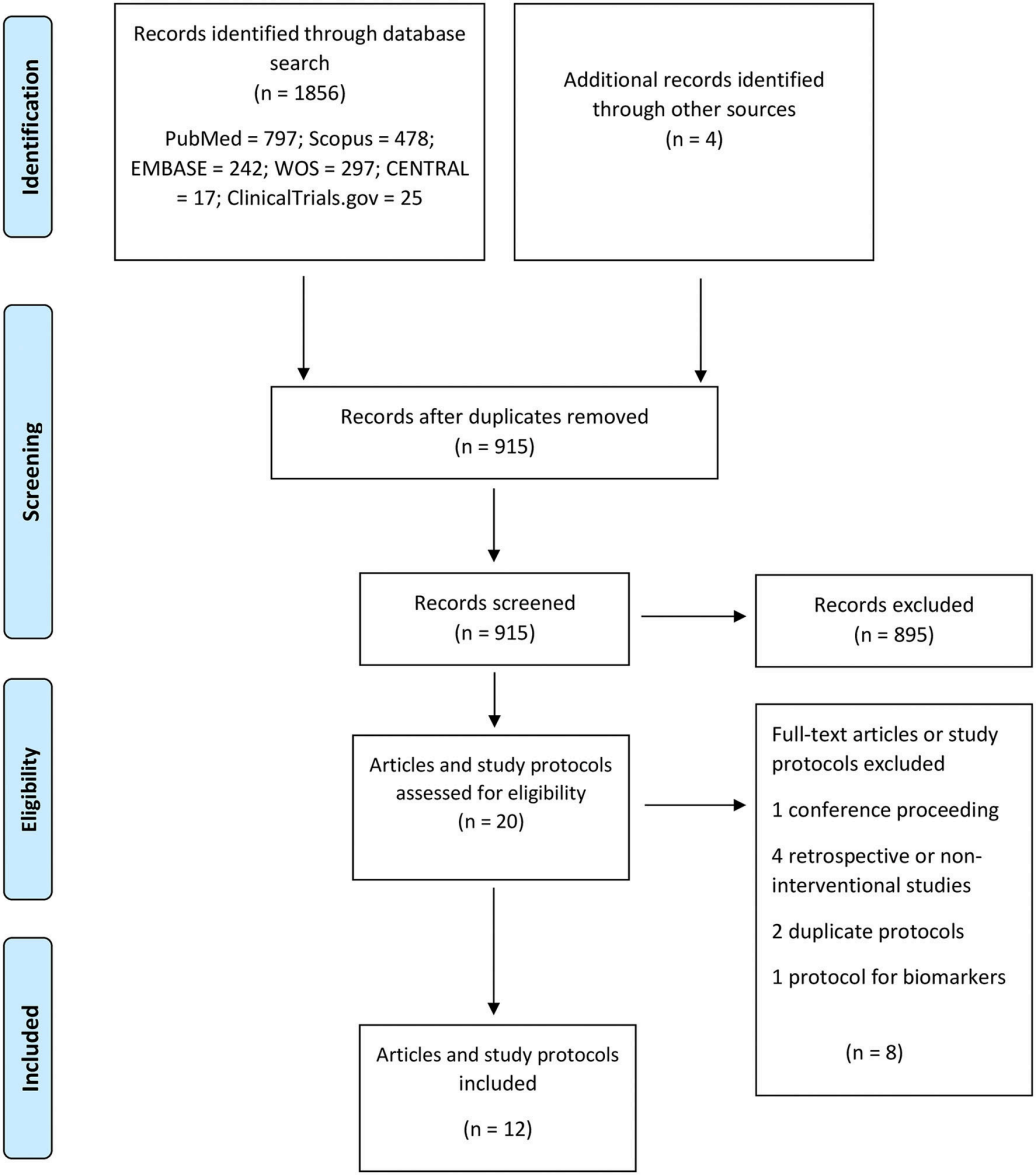
PRISMA flow diagram for the included studies.

Out of 915 published papers and registered study protocol, six papers and six study protocols met our inclusion criteria and were selected for data extraction (Figure 3). The summary of the included papers and study protocols are shown in Tables 1, 2, respectively.

**Table 1:** Summary of the published interventional studies.

**Author location [Reference no.]**	**Number of patients vs. controls**	**Immune functional assay [Reference no.]**	**Study design**	**Summary of methods**	**Main Findings**	**Imitations**	**The overall risk of bias**
Ravaioli et al. (39), Italy	Adult liver transplant recipients (*n* = 202 including 102 patients in the intervention and 100 in the control group)	ImmuKnow®	Randomized clinical trial	In the intervention group, the tacrolimus dose decreased by 25% when the ImmuKnow® result was compatible with a low immune response (<130 ng/mL ATP) and increased by 25% when the ImmuKnow® result was compatible with a robust immune response (>450 ng/mL ATP). ImmuKnow® was done before transplantation, immediately after the transplantation, on the first day after, weeks 1 to 4, 6, and 8, and months 3 to 6, 9, and 12 post-transplantation. In the control group, the tacrolimus dose was tapered according to the standard practice in the center.	Liver transplant recipients with a higher model for end-stage liver disease (MELD) score had lower ImmuKnow® results. One-year survival was significantly higher in the intervention group. The rate of infection episodes later than 14 days post-transplantation was lower in the intervention group than in controls. Recipients in the control group had a higher risk of hospital admission due to infection and a longer duration of admission than the intervention group. Median through-level of tacrolimus was significantly lower in the intervention group at third, sixth, and twelfth months post-transplantation than controls. ImmuKnow® was correlated with the presence of infection during the first 6 months post-transplantation, while tacrolimus through-level was only correlated with the presence of infections during the first-month post-transplantation.	Only liver transplant recipients included.	Some concerns
Kim et al. (40), Korea	Adult kidney and/or pancreas transplant recipients (*n* = 784) 521 out of 784 had negative or indeterminate T-SPOT^®.^TB results, while 262 had positive T-SPOT^®.^TB results. 263 T-SPOT®.TB positive transplant recipients randomly assigned in the intervention (*n* = 131) or control (*n* = 132) groups.	T-SPOT^®.^TB	Randomized clinical trial	The kidney and or pancreas transplant recipients who had positive T-SPOT^®.^TB but had no clinical or radiologic evidence of latent tuberculosis infection, randomized in intervention or control group. Patients in the intervention group received 9 months of isoniazid, which was started after transplantation. The control group did not receive isoniazid and only were followed up.	In a median follow up of 1.8 years post-transplantation, 7 out of 784 (0.9%) transplant recipients developed tuberculosis. None of the transplant recipients in the interventional group developed tuberculosis, while 3 out of 132 (2%) in the control group and 4 out of 521 (1%) in the IGRA negative group developed tuberculosis.	Insufficient statistical power.	Some concerns
Westall et al. (41), Australia	Adult lung transplant recipients (*n* = 118)	QuantiFERON®-CMV assay	Randomized clinical trial	Adult lung transplant recipients who were at high risk of CMV infection (D+/R–) or reactivation (D+/R+), received antiviral prophylaxis for 5 months after transplantation, at the end of the fifth month, QuantiFERON®-CMV assay was done and transplant recipients categorized according to the results. Antiviral prophylaxis discontinued in SOT recipients who had positive QuantiFERON®-CMV assay (≥0.2 IU/mL IFN-γ) (Control group, *n* = 36). In the intervention group (*n* = 82), antivirals postponed for 3 months, and this process repeated at the end of the eighth and eleventh months post-transplantation. Regardless of the QuantiFERON®-CMV assay results, prophylaxis discontinued at the end of the eleventh month.	CMV infection within the lung allograft was observed in 21 out of 36 (58%) of controls in comparison with 30 out of 82 (37%) in the intervention group (*P* = 0.03). The incidence of viremia was 6 (16.7%) in controls and 3 (3.7%) in the intervention group (*P* = 0–02).	Only lung transplant recipients included.	Some concerns
Poglajen et al. (42), Slovenia	Adult heart transplant recipients (*n* = 154)	QuantiFERON®-CMV assay	Non-randomized clinical trial	All the heart transplant recipients received antiviral prophylaxis for 100 days post-transplantation. CMV PCR was done on the day 100 post-transplantation, and SOT recipients who had negative CMV PCR were categorized into QuantiFERON®-CMV assay guided or standard of care groups. In the QuantiFERON®-CMV assay guided (the intervention group, 41 out of 154) a QuantiFERON®-CMV assay was done and antiviral prophylaxis discontinued if the result was positive. However, if the result was negative, QuantiFERON®-CMV assay repeated every 100 days until the assay became positive, and at that time, antiviral prophylaxis was discontinued. In the standard of care (the control group, 113 out of 154) antiviral prophylaxis discontinued after 100 days. Participants were followed for 1 year after discontinuation of antiviral prophylaxis. Prior to transplantation, 81, 17, and 2% of recipients in the intervention and 84, 12, and 4% in the control groups were D±/R+, D+/R–, and D–/R–, respectively.	The mean duration of antiviral prophylaxis was longer in the intervention group than the control group (155 ± 102 days vs. 104 ± 48 days, *p* < 0.05). During 12 months of follow up, the intervention group had lower rates of CMV infection than the control (5 vs. 19%, *p* = 0.03).	Non-randomized and single-center study.	Serious
Kumar et al. (43), Canada	27 Adult SOT recipients (7 kidney, ten liver, six lung, and four combined SOT recipients) (14 QuantiFERON®-CMV positive and 13 QuantiFERON®-CMV negative)	QuantiFERON®-CMV assay	Non-randomized clinical trial	Antiviral treatment started in SOT recipients with the first documented episode of CMV infection according to the standard clinical care. At the end of treatment, a blood sample was taken, and the QuantiFERON®-CMV assay was done. Antiviral discontinued in SOT recipients with positive QuantiFERON®-CMV assay (≥0.2 IU/mL IFN-γ). Otherwise, antivirals were postponed for 2 months (secondary prophylaxis). SOT recipients monitored with CMV PCR, on 2-weeks intervals, and up to 3 months after completion of the treatment.	All the 27 SOT recipients responded to antiviral therapy, and CMV viral load was undetectable in a median of 33 days after transplantation. Out of the 27 SOT recipients, 14 recipients were QuantiFERON-CMV assay positive, and 13 QuantiFERON®-CMV assay negative. Nine out of the 13 QuantiFERON®-CMV negative and 1 of the 14 QuantiFERON®-CMV positive SOT recipients had CMV recurrence (*p* = 0.001).	Small sample size. It only included asymptomatic patients with CMV viremia or mild to moderate disease.	Moderate
Jarque et al. (44), Spain	Kidney transplant recipients (*n* = 160)	T-SPOT.CMV assay	Randomized clinical trial	Kidney transplant recipients who were anti-CMV Ab D+/R+ enrolled in this study. T-SPOT®.CMV (IE-1 specific) assay was done prior to transplantation, and according to the results, SOT recipients were allocated into the Group A (low risk, *n* = 103) or Group B (high risk, *n* = 57). The cut-off for low and high-risk SOT recipients was 20 spots per 300,000 PBMCs. SOT recipients in both group A and B, underwent randomization to receive either antiviral prophylaxis	During 12 months follow up, 57 out of 160 (36%) and 9 out of 160 (6%) of SOT recipients developed CMV infection and disease, respectively. High risk SOT recipients who received preemptive therapy had higher rates of CMV infection (73 vs. 44%, *p* = 0.013), CMV infection required treatment (53 vs. 19%, *p* = 0.001) and CMV disease (20 vs. 4%, *p* = 0.028) than low risk SOT recipients who received preemptive therapy.	Only D+/R+ kidney transplant recipients included.	Some concerns
				(for 3 months) or preemptive antiviral therapy and followed up for 12 months. In addition, T-SPOT®.CMV was done 15 days post-transplantation in both groups.	When high and low-risk SOT recipients who received prophylactic antiviral were compared, rates of CMV infection was higher in the high-risk group (33 vs. 4%, *p* = 0.003). While the significance in the rate of CMV infection required treatment, was borderline (19 vs. 4%, *p* = 0.056) and CMV disease (4 vs. 0%, *p* = NA) was not significantly different. Risk stratification of SOT recipients according to the T-SPOT®.CMV values 15 days post-transplantation, was more accurate than the risk stratification according to the T-SPOT®.CMV values prior to the transplantation.		

**Table 2:** Summary of the registered interventional study protocols.

**Code, location Start year [Reference no.]**	**Number of patients vs. controls**	**Detection method [Reference no.]**	**Study design**	**Summary of methods**	**Aims of the study**
NCT01424345, USA, 2011, NIH (45), (This trial finished in 2012, but the results are not accessible).	Adult kidney transplant recipients (Estimated number of participants is 40)	ImmuKnow®	Randomized controlled trial	Adult kidney transplant recipients will be included in this study. The dosage of immunosuppressive agents in the control group will be adjusted according to the current laboratory results. In the intervention group, the dosage of immunosuppressive agents will be adjusted according to the results of ImmuKnow and the routine post-transplant lab results. Both of the groups will be followed up for 12 months after transplantation.	To define the proportion of infection and rejection during the first year after transplantation. To define the quality of life of the kidney transplant recipients one-year post-transplantation. To define allograft function and graft and recipient survival in the first year post-transplantation.
NCT03699254, Spain, 2011, Paez-Vega et al. (46)	Adult lung transplant recipients (Estimated number of participants is 150 including 75 in the interventional and 75 in the control group)	QuantiFERON®-CMV assay	Phase III randomized, multicenter, non-inferiority clinical trial	Adult lung transplant recipients who are at risk of CMV reactivation (–/R+) will include in this study. In the control group, lung transplant recipients will receive anti-CMV prophylaxis for 6 months post-transplantation. From the end of the sixth month up to the end of the twelfth month, episodes of CMV reactivation will be treated by antivirals. In the intervention group, lung transplant recipients will receive antiviral prophylaxis for 3 months after transplantation. QuantiFERON®-CMV will be done at the end of the third month, and antiviral prophylaxis will be discontinued if the QuantiFERON®-CMV is positive (≥0.2 IU/mL IFN-γ). If the QuantiFERON®-CMV is negative, antivirals prophylaxis will be continued for one additional month, and this process will be repeated monthly up to the end of the twelfth month after transplantation. All the recipients will follow for 18 months or more post-transplantation for any evidence of CMV infection.	To define the efficacy of the immune-guided antiviral prophylaxis for the prevention of CMV disease in lung transplant recipients (Intervention group). To define the efficacy of the universal antiviral prophylaxis for the prevention of CMV disease in lung transplant recipients (Control group). To compare interventional and control groups. To define a new cut-off for the QuantiFERON®-CMV assay, if applicable.
CN-01898092, Czech Republic, 2018, ILTS (47)	Adult kidney transplant recipients (Estimated number of participants is 150)	Quantiferon®-CMV assay	Phase IV randomized, non-inferiority clinical trial	Prior to transplantation adult kidney transplant recipients who are D+/R–, D+/R+, or D-/R+ will be randomized to one of the QuantiFERON®-CMV guided (intervention group) or standard protocol guided (control group) pre-emptive therapy. Weekly quantitative CMV PCR will be done during the first 4 weeks post-transplantation. Three weeks post-transplantation a second QuantiFERON®-CMV assay will be done in the intervention group and QuantiFERON®-CMV positive SOT recipients (CMV stimulation ≥0.2 IU/mL plus mitogen stimulation ≥0.5 IU/mL) will be followed with quantitative CMV PCR on months 2, 3, 4, 5, 6, 7, 8, 9, 10, 11, 12, 15, 18, 21, and 24. QuantiFERON®-CMV negative (CMV stimulation <0.2 IU/mL plus mitogen stimulation ≥0.5 IU/mL) and indeterminate (CMV stimulation <0.2 IU/mL plus mitogen stimulation <0.5 IU/mL) patients, as well as SOT recipients in the control group, will be undergone weekly CMV PCR up to the month 4 post-transplantation. Thereafter, CMV PCR will be done on months 5, 6, 7, 8, 9, 10, 11, 12, 15, 18, 21, and 24. At any point, the CMV viral load more than 1,000 IU/mL will be treated with valganciclovir or gancyclovir per protocol. All patients will be followed up to 4 years after transplantation or until death.	To define the cumulative incidence of CMV infection (DNAemia) with a viral load of ≥2,000 IU/mL defined by positive PCR for CMV DNA in whole blood up to 12 months post-transplantation. To define the cumulative incidence of CMV disease (defined by clinical symptoms + presence of CMV DNAemia by quantitative PCR CMV DNA test). To define the graft and patients' survival and complications up to 36 months post-transplantation.
NCT02784756, Canada, 2016, NIH (48)	Adult kidney, kidney-pancreas, liver, or heart transplant recipient (Estimated number of participants is 200)	Quantiferon®-CMV assay	Phase III clinical trial with a single arm	Adult SOT recipients who are D+/R– or –/R+ and receive antithymocyte globulin induction therapy will include in this study. The duration of antiviral prophylaxis will define according to the results of Quantiferon®-CMV assay in specific time points during the study. More details are not accessible.	To use Quantiferon®-CMV assay as a guide for the duration of primary CMV prophylaxis in SOT recipients. To define the number of SOT recipients with symptomatic CMV disease (Tissue invasive or viremia) during the first year after transplantation.
NCT03123627, Spain, 2016(49)	Adult kidney transplant recipients (Estimated number of participants is 105)	Quantiferon®-CMV assay	Phase III randomized Clinical Trial	Adult kidney transplant recipients who are CMV seropositive and Quantiferon®-CMV assay reactive at the time of transplantation and receive antithymocyte globulin induction therapy will be included in this study. The control group will receive a fixed duration of 3 months of antiviral prophylaxis after transplantation. The intervention group will receive antiviral prophylaxis and will be monitored with Quantiferon®-CMV assay at the days +15, +30, and +60 post-transplantation. The antiviral will be discontinued if the Quantiferon®-CMV assay is positive. Otherwise, antiviral will be continued up to the day +90 post-transplantation. Both groups will be followed 12 months post-transplantation for evidence of CMV disease.	To define the incidence of CMV disease in kidney transplant recipients at 12 months after transplantation. To define the predictive value of Quantiferon®-CMV assay for the duration of anti-CMV antiviral prophylaxis.
NCT02538172, Switzerland, 2015, NIH (50)	Adult kidney and liver transplant recipients (Estimated number of participants is 200)	T-Track® CMV assay and Quantiferon-CMV® assays	Randomized controlled trial	Adult SOT recipients who are D+/R– or –/R+ and receive antithymocyte globulin induction therapy will be included in this study. The control group will receive a fixed duration of 3 or 6 months of antiviral prophylaxis. In the intervention group, SOT recipients will receive CMV antiviral prophylaxis and will be monitored with two different assays every 4 weeks from the second month after transplantation. Antiviral prophylaxis will be discontinued in T-Track® CMV assay positive patients and will be continued until the maximal duration of prophylaxis (3–6 months). After discontinuation of the antiviral prophylaxis, SOT recipients in both groups will be followed up in for evidence of CMV replication up to 12 months after transplantation. If viral replication detects by PCR, standard treatment will be done according to local guidelines.	To use CMI as a guide for the duration of primary CMV prophylaxis in SOT recipients. To define the incidence of CMV infection and CMV viremia in SOT recipients during the first year after transplantation. To define graft survival during the study, follow up.

### ImmuKnow® Guided Adjustment of Immunosuppressive and Anti-infective Agents in SOT Recipients

#### Liver Transplant Recipients

In a randomized trial of 202 adult liver transplant recipients, SOT recipients were assigned to the intervention (100 out of 202) or control groups (102 out of 202) and were followed for 1-year post-transplantation. ImmuKnow® was used to guide the dosage of tacrolimus in the intervention group (39). The tacrolimus dosage was decreased by 25% when the ATP value was lower than 130 ng/mL and increased by 25% when the ATP value was higher than 450 ng/mL (39). In the control group, the tacrolimus dosage was adjusted according to the tacrolimus trough level. Tacrolimus trough level on month 3, 6, and 12 post-transplantation was lower in the intervention group than in the controls. SOT recipients in the intervention group had lower incidence of bacterial (32 vs. 46%, *p* < 0.05) and fungal (2 vs. 11%, *p* < 0.05) infections during the first year post-transplantation (39). However, the incidence of viral infections (22 vs. 23%) was similar. Lower ATP levels were associated with the presence of any infection during the first 6 months post-transplantation. In contrast, the tacrolimus trough level was only correlated with the presence of infection during the first month but not with infection in the last 11 months of the first year post-transplantation (39).

#### Kidney Transplant Recipients

Among registered clinical trials, we only found one open-label randomized control trial that used ImmuKnow®, and this trial was registered in 2011 and finished in 2012 (45). However, we could not find any results or published articles related to the clinical trial. No other ongoing or published interventional studies have used ImmuKnow® to guide dosage of anti-infective agents in adult SOT recipients.

### QuantiFERON®-TB Gold and/or T-SPOT®.TB Guided Adjustment of Immunosuppressive and Anti-infective Agents in SOT Recipients

#### Kidney Transplant Recipients

T-SPOT®.TB assay was used to decide when to initiate anti-tuberculosis prophylaxis in a randomized clinical trial of 784 adult kidney and/or pancreas transplant recipients in south Korea which is an intermediate-TB-burden country (40). SOT recipients with positive T-SPOT®.TB (263 out of 784), who did not have any clinical or radiologic evidence of active tuberculosis, were randomly assigned to the intervention (131 out of 263) or control (132 out of 263) groups. SOT recipients with negative or indeterminate T-SPOT®.TB results were followed in a parallel arm (521 out of 784) of the study. SOT recipients in the intervention group received 9 months of isoniazid as anti-tuberculosis prophylaxis, while controls, as well as patients in the parallel arm, did not receive isoniazid. In a median follow up of 1.8 years, none of the SOT recipients in the intervention group developed tuberculosis, while 2% of the controls (incidence rate of 1.22 per 100 person-years) and 0.8% of the SOT recipients in the parallel-arm (incidence rate of 0.43 per 100 person-years) were diagnosed with tuberculosis (40).

We could not find any other published interventional studies or study protocols that used QuantiFERON®-TB Gold for dose adjustment of immunosuppressive or anti-infective agents in SOT recipients.

### QuantiFERON®-CMV, T-Track® CMV, or T-SPOT®.CMV Guided Adjustment of Immunosuppressive and Anti-infective Agents in SOT Recipients

#### Heart Transplant Recipients

A recent study used QuantiFERON®-CMV to adjust the duration of anti-CMV antiviral (valganciclovir) prophylaxis in a non-randomized clinical trial of 154 heart transplant recipients (42). Prior to transplantation, more than 80% of SOT recipients were CMV seropositive while had seropositive or seronegative donors. All the SOT recipients received antiviral prophylaxis for 100 days post-transplantation. CMV polymerase chain reaction (PCR) was done on the day 100 post-transplantation, and SOT recipients who had negative CMV PCR were categorized into QuantiFERON®-CMV assay guided or standard of care groups. In the QuantiFERON®-CMV assay guided (the intervention group, 41 out of 154) a QuantiFERON®-CMV assay was done and antiviral prophylaxis discontinued if the result was positive. However, if the result was negative, QuantiFERON®-CMV assay repeated every 100 days and antiviral prophylaxis continued until the assay became positive, and at that time, antiviral prophylaxis was discontinued. In the standard of care (the control group, 113 out of 154) antiviral prophylaxis discontinued after 100 days. Participants were followed for 1 year after discontinuation of antiviral prophylaxis. Using QuantiFERON®-CMV, the duration of anti-CMV antiviral prophylaxis was longer (155 ± 102 days vs. 104 ± 48 days, *p* < 0.05), but the rate of CMV infection (5 vs. 19%, *p* = 0.03) was lower in the intervention group (42).

#### Lung Transplant Recipients

In another clinical trial of 118 CMV seropositive lung transplant recipients, QuantiFERON®-CMV was used to guide the duration of antiviral prophylaxis (41). All the SOT recipients received antiviral prophylaxis for 5 months post-transplantation. QuantiFERON®-CMV was done at the end of the fifth month, and antiviral prophylaxis discontinued in SOT recipients who had positive results (control group). In SOT recipients who had negative QuantiFERON®-CMV, antiviral prophylaxis was continued, and viral PCRs were monitored regularly up to the time point when the QuantiFERON®-CMV result converted to positive (Interventional group). The maximum duration of antiviral prophylaxis in SOT recipients who had negative QuantiFERON®-CMV results was 11 months. The CMV infection in the lung allograft was defined as CMV viral load of more than 600 copies/mL in bronchoalveolar lavage (BAL). The incidence of CMV viremia (23 vs. 33%, *p* = ns) and CMV infection in the lung allograft (37 vs. 58%, *p* = 0.03) was lower in the intervention group than in the control group. In other words, QuantiFERON®-CMV guided prophylaxis favored a lower incidence of CMV in lung allograft (i.e. BAL) but not CMV viremia. Although, the incidence of severe lung CMV infection (CMV viral load >10,000 copies in BAL) did not differ between the two arms (11 vs. 11%, *p* = ns) (41).

Several trails of QuantiFERON®-CMV in SOT recipients are underway. An ongoing phase III clinical trial on an estimated number of 150 lung transplant recipients was registered in 2019. In this clinical trial, researchers plan to use QuantiFERON®-CMV guided anti-CMV prophylaxis to reduce the duration of antiviral prophylaxis (46).

#### Kidney Transplant Recipients

A phase III clinical trial with an estimated number of 105 CMV-seropositive kidney transplant recipients plans to assess QuantiFERON®-CMV guided anti-CMV prophylaxis (49). A phase IV randomized clinical trial registered in 2018 with an estimated number of 150 adult kidney transplant recipients aims to use QuantiFERON®-CMV as a guide for preemptive therapy of the CMV infection (47).

A recent multicenter double-blind randomized clinical trial included adult kidney transplant recipients who were anti-CMV antibody donor positive / recipient positive prior to transplantation (44). This clinical trial used T-SPOT®.CMV and assigned SOT recipients into the interventional groups. T-SPOT®.CMV (IE-1 specific) assay was done prior to transplantation, and according to the results, SOT recipients were allocated into the low-risk or high-risk groups. The cut-off to discriminate low- and high-risk SOT recipients were 20 spots per 300,000 PBMCs. Both low- and high-risk SOT recipients underwent randomization to receive either anti-CMV prophylaxis (for 3 months) or preemptive antiviral therapy (44). In addition, T-SPOT®.CMV was done 15 days post-transplantation in both groups. During one-year follow-up post-transplantation, 57 out of 160 (36%) and 9 out of 160 (6%) of SOT recipients developed CMV infection and disease, respectively. High risk SOT recipients who received preemptive therapy had higher rates of CMV infection (73% vs. 44%, p = 0.013), CMV infection that required treatment (53 vs. 19%, *p* = 0.001) and CMV disease (20 vs. 4%, *p* = 0.028) than low-risk SOT recipients who received preemptive therapy. When low- and high-risk SOT recipients who received prophylactic antiviral treatment were compared, rates of CMV infection was higher in the high-risk group (33 vs. 4%, *p* = 0.003) (44).

#### Heart, Lung-, Liver-, Kidney- and Pancreas Transplant Recipients

Other than primary prophylaxis, QuantiFERON®-CMV has been used to guide the duration of secondary prophylaxis in a group of 27 SOT recipients including lung, liver, and kidney transplant recipients (43). Antiviral treatment started in SOT recipients with the first documented episode of CMV infection according to the standard of clinical care. At the end of treatment, the QuantiFERON®-CMV assay was done. Antiviral treatment was discontinued in SOT recipients with positive QuantiFERON®-CMV assay (≥0.2 IU/mL IFN-γ). Otherwise, oral valganciclovir or intravenous ganciclovir were continued for 2 months (secondary prophylaxis). SOT recipients were monitored with CMV PCR with 2-weeks intervals, and up to 3 months after completion of the treatment. SOT recipients who had negative QuantiFERON®-CMV, and received secondary prophylaxis, had a higher rate of recurrence during follow up compared to QuantiFERON®-CMV positive recipients (9 out of the 13 vs. 1 out of the 14, *p* = 0.001). The study concluded that in the SOT recipients who finished their treatment course and had a positive QuantiFERON®-CMV, it is reasonable to discontinue secondary antiviral prophylaxis (43).

A phase III clinical trial registered in 2016, aims to use QuantiFERON®-CMV guided anti-CMV prophylaxis in an estimated number of 200 adult kidney-, pancreas-, liver-, or heart transplant recipients who received induction therapy with antithymocyte globulin (48). An open-label clinical trial was registered in 2015 and planned to use T-Track® CMV to guide the duration of primary CMV antiviral prophylaxis in kidney-or liver-transplant recipients. The estimated number of SOT recipients to be included in this clinical trial was 200 and the status of the clinical trial is recruiting and results have not communicated yet (50).

### TruCulture® and Adjustment of Immunosuppressive and Anti-infective Agents in SOT Recipients

TruCulture® is a relatively new IFA in comparison with ImmuKnow® and IGRAs. Hence, it has not been used in clinical trials on SOT recipients yet. We could not find any published or registered clinical trial that used TruCulture® in SOT recipients.

### Risk of Bias

**Figure 4:**
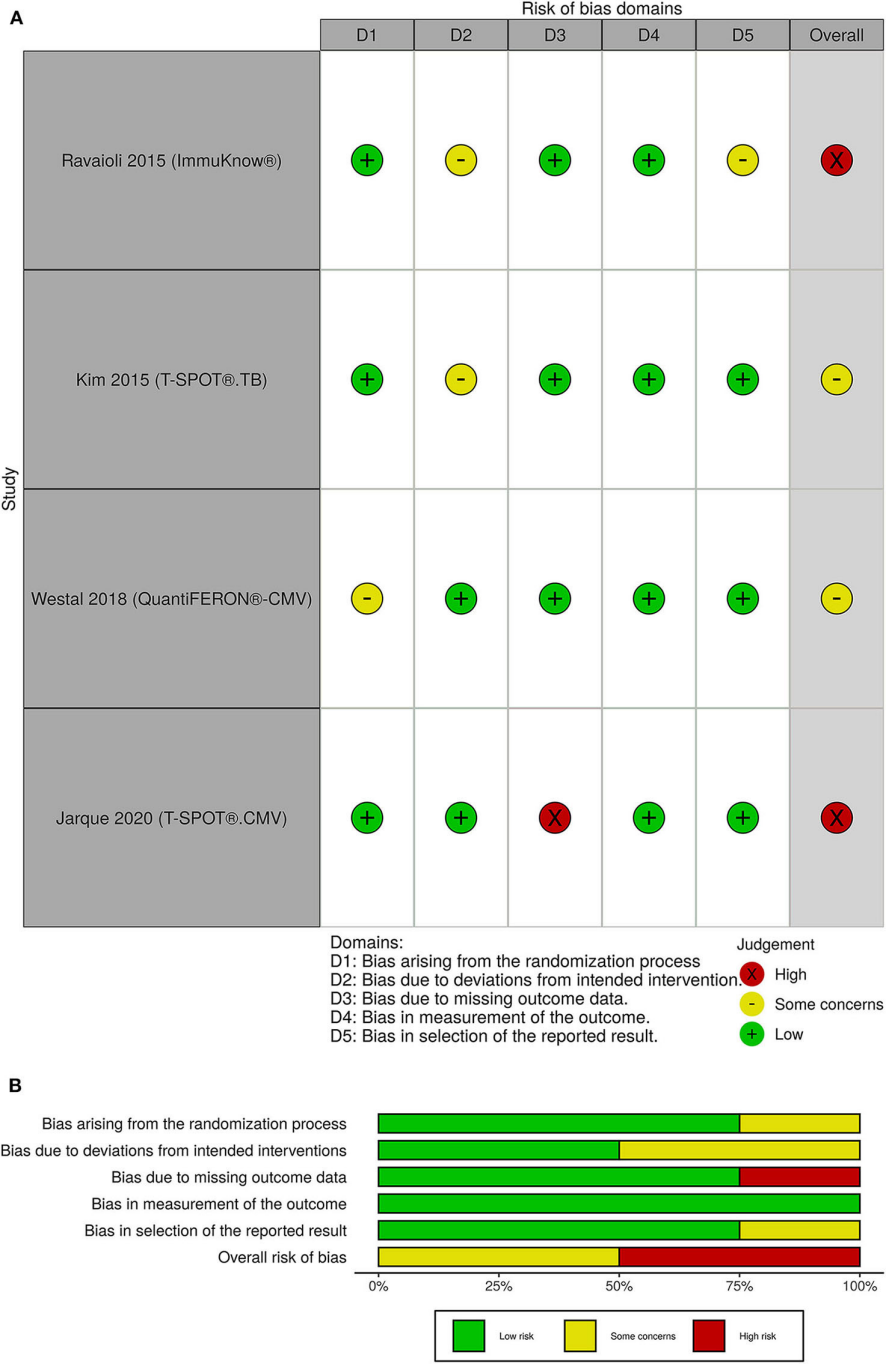
**(A)** Traffic light plots; **(B)** weighted bar plots for randomized clinical trials. The overall risk of bias was with some concerns for 2 out of 4 randomized clinical trials. The other 2 randomized clinical trials had a high risk of bias.

**Figure 5:**
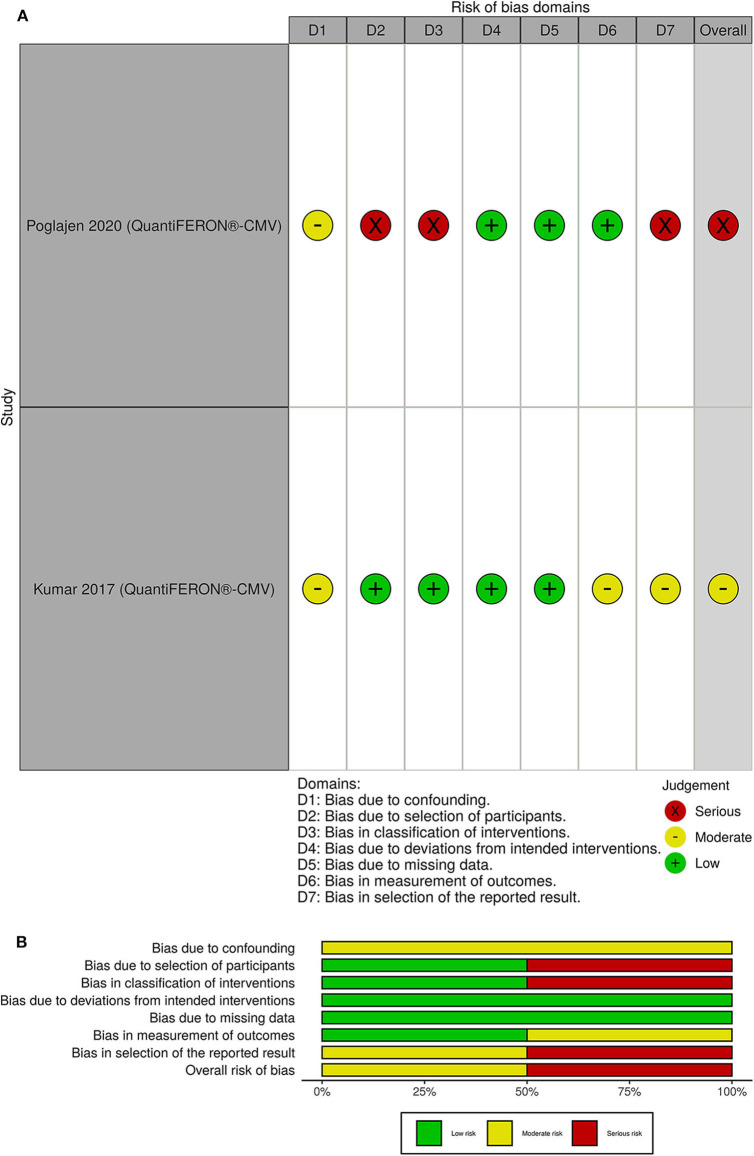
**(A)** Traffic light plots; **(B)** weighted bar plots for non-randomized clinical trials. One of the non-randomized clinical trials had a serious overall risk of bias and the other one had a moderate risk of bias.

The overall risk of bias was with some concerns for 2 out of 4 randomized clinical trials. The other 2 randomized clinical trials had a high risk of bias (Figure 4). One of the non-randomized clinical trials had a serious overall risk of bias and the other one had a moderate risk of bias (Figure 5).

## Discussion

In a systematic review, we found only six published papers and six registered protocols describing interventional studies aiming to investigate the use of IFAs for adjustment of the immunosuppressive or anti-infective agents in SOT recipients. Four out of the six published clinical trials used one of the commercially available IGRA-CMVs (41–44), and five out of the six registered protocols planned to use the IGRA-CMVs for adjustment of the antiviral prophylaxis or preemptive therapy (46, 48–50).

Infections and drug-related complications are highly prevalent and affect both the survival and quality of life of the SOT recipients (51–54). Only one published interventional study used ImmuKnow® to adjust the dosage of immunosuppression in liver transplant recipients (39). Using ImmuKnow® as a guide to dosing of tacrolimus subsequently decreased bacterial and fungal infections and improved survival in SOT recipients (39). However, it was a single-center study, only liver transplant recipients were included and there were some concerns for risk of bias due to deviation from intended intervention and reported results (39). ImmuKnow® uses PHA as a stimulant and measures the ATP that is produced by CD4+ T lymphocytes. Previous meta-analyses have not reached a consistent conclusion on the routine use of ImmuKnow® for prediction of infection in SOT recipients (55, 56). A meta-analysis included ten studies and showed that ImmuKnow® could be used for the prediction of infections in liver transplant recipients (55). The other meta-analysis included six studies in kidney transplant recipients but the results did not support the role of ImmuKnow® for the prediction of infections (56). It should be noted that the study that we included in our systematic review was not part of the previous meta-analyses (55, 56). Other than the need for further clinical trials to define the safety and feasibility of ImmuKnow® in SOT recipients, we need studies to define the most appropriate ATP cut-off for discrimination of SOT recipients who are at risk of infections (16).

One randomized clinical trial has used an IGRA-TB (T-SPOT®.TB) to guide the use of anti-tuberculosis prophylaxis in SOT recipients (40). In the mentioned study, tuberculosis developed in SOT recipients who did not receive anti-tuberculosis prophylaxis regardless of the T-SPOT®.TB results, although the incidence rate was higher in T-SPOT®.TB positive SOT recipients. The study was well-designed and the overall risk of bias was low (40). It is reported that IGRA-TBs have a high rate of indeterminate results (23), and underestimate the real burden of latent tuberculosis in SOT recipients (48). Therefore, according to the current evidence, it is not possible to decide for or against the initiation of anti-tuberculosis prophylaxis in SOT recipients who have negative or indeterminate T-SPOT®.TB results.

Despite prophylaxis and preemptive antiviral strategies, CMV remains an important cause of post-transplantation infections (51, 57). Moreover, CMV is among the main causes of early graft loss, mortality, and morbidity in SOT recipients (58). Given the importance and burden of CMV infections and accessibility of CMV-specific and commercially available IFAs, it is justifiable that most of the studies that we found targeted CMV.

The feasibility of the IGRA-CMVs for adjustment of the duration of primary or secondary CMV antiviral prophylaxis has been shown in SOT recipients (41–43). It seems to be possible to reduce the duration of primary or secondary CMV antiviral prophylaxis in SOT recipients who are QuantiFERON®-CMV positive post-transplantation (41–43), although the overall risk of bias for current clinical trials are moderate to high and solid evidence preferentially from larger well-designed studies including all types of transplanted solid organs are warranted. Other than QuantiFERON®-CMV, it is possible to use T-SPOT®.CMV for risk stratification of the SOT recipients (44). However, the best cut-off point for discrimination of the positive and negative IGRA-CMV results is not a fixed value. One of the ongoing clinical trials plans to define a new cut-off for QuantiFERON®-CMV in lung transplant recipients (37). The stimulating antigen, the method for measuring the response, and timing of the assessment can significantly affect the results and the cut-off value (44, 59).

In this systematic review, we used a wide range of keywords to find all the available evidence regarding the use of IFAs to guide the dosing of immunosuppressants or anti-infective agents in SOT recipients. It should be mentioned that in the current guidelines there are different clinical indications for use of the IGRA-CMV and IGRA-TB in SOT recipients. IGRA-TB is an assays used for risk assessment of latent TB prior to transplantation (60). Although IGRA-CMV can be used for risk assessment of CMV infection prior to transplantation, IGRA-CMV is mainly used for assessment of risk of CMV reactivation post-transplantation (58). We searched four medical research databases and two clinical trial registries. Nevertheless, some of the ongoing clinical trials might be registered in local or regional registries, and we may have missed such protocols in our systematic review.

In conclusion, our systematic review supports the role of IGRA-CMVs for adjustment of the duration of anti-CMV antiviral prophylaxis in SOT recipients. We do not have enough evidence regarding the routine use of the other T cell mediated IFAs in guiding duration and dosage of immunosuppressive agents or anti-infective agents in SOT recipients to make conclusions regarding the clinical utility. Currently, several ongoing clinical trials are underway, but large, randomized clinical trials including other T cell mediated IFAs and all types of transplanted solid organs are needed to evaluate the role of IFAs in guiding immunosuppressive and anti-infective therapy in SOT recipients.

## Data Availability Statement

The original contributions generated for this study are included in the article/supplementary material, further inquiries can be directed to the corresponding author/s.

## Author Contributions

OR, DM, SS, AR, MP, FG, SO, and SN designed the study. OR performed the initial search and wrote the first draft of the manuscript, DM and AK cross-checked the referenced articles. OR and DM summarized the articles. SS, AR, MP, FG, SO, and SN supervised the review. OR, DM, AK, SS, AR, MP, FG, SO, and SN revised and commented on the manuscript. All authors read and approved the final version of the manuscript.

## Conflict of Interest

OR received a grant from The Research Foundation of Rigshospitalet related, and a grant from A.P. Møller Fonden not related to this work; AK received a grant from The Danish Heart Foundation and a traveling grant from Gilead not related to this work; MP received a grant from Roche, non-financial support from Boehringer Ingelheim, personal fees from Mallinckrodt, Novartis, and Astra-Zeneca not related to this work; FG received personal fees from Abbott, Novartis, Boehringer-Ingelheim, Orion Pharma, Pfizer, Astra-Zeneca and had other financial relationship with Corvia not related to this work; SN received a grant from Novo Nordisk Foundation. The remaining authors declare that the research was conducted in the absence of any commercial or financial relationships that could be construed as a potential conflict of interest.

## References

[1] Black CK Termanini KM Aguirre O Hawksworth JS Sosin M. Solid organ transplantation in the 21st century. Ann Transl Med. (2018) 6:409. 10.21037/atm.2018.09.68 30498736 PMC6230860

[2] Ling X Xiong J Liang W Schroder PM Wu L Ju W Can immune cell function assay identify patients at risk of infection or rejection? A meta-analysis. Transplantation. (2012) 93:737–43. 10.1097/TP.0b013e3182466248 22357178

[3] Mells G Neuberger J. Long-term care of the liver allograft recipient. Semin Liver Dis. (2009) 29:102–20. 10.1055/s-0029-1192059 19235663

[4] Drabe CH Sorensen SS Rasmussen A Perch M Gustafsson F Rezahosseini O. Immune function as predictor of infectious complications and clinical outcome in patients undergoing solid organ transplantation (the ImmuneMo:SOT study): a prospective non-interventional observational trial. BMC Infect Dis. (2019) 19:573. 10.1186/s12879-019-4207-9 31269923 PMC6609391

[5] Enderby C Keller CA. An overview of immunosuppression in solid organ transplantation. Am J Manag Care. (2015) 21:s12–23. 25734416

[6] Holt CD. Overview of immunosuppressive therapy in solid organ transplantation. Anesth Clin. (2017) 35:365–80. 10.1016/j.anclin.2017.04.001 28784214

[7] Hand J Patel G. Antimicrobial stewardship in transplant patients. Curr Opin Organ Transplant. (2019) 24:497–503. 10.1097/MOT.0000000000000661 31145159

[8] Weston MW Rinde-Hoffman D Lopez-Cepero M. Monitoring cell-mediated immunity during immunosuppression reduction in heart transplant recipients with severe systemic infections. Clin Transplant. (2020) 34:e13809. 10.1111/ctr.13809 32003048

[9] Mouton W Vega CA Boccard M Bartolo F Oriol G Lopez J. Towards standardization of immune functional assays. Clin Immunol. (2019) 210:108312. 10.1101/718627 31760096

[10] Kowalski R Post D Schneider MC Britz J Thomas J Deierhoi M. Immune cell function testing: an adjunct to therapeutic drug monitoring in transplant patient management. Clin Transplant. (2003) 17:77–88. 10.1034/j.1399-0012.2003.00013.x 12709071

[11] Albert-Vega C Tawfik DM Trouillet-Assant S Vachot L Mallet F Textoris J. Immune functional assays, from custom to standardized tests for precision medicine. Front Immunol. (2018) 9:2367. 10.3389/fimmu.2018.02367 30386334 PMC6198655

[12] Ruhwald M de Thurah L Kuchaka D Zaher MR Salman AM Abdel-Ghaffar A-R. Introducing the ESAT-6 free IGRA, a companion diagnostic for TB vaccines based on ESAT-6. Sci Rep. (2017) 7:45969. 10.1038/srep45969 28387329 PMC5384086

[13] Han SH. Immunological prediction of cytomegalovirus (CMV) replication risk in solid organ transplantation recipients: approaches for regulating the targeted anti-CMV prevention strategies. Infect Chemother. (2017) 49:161–75. 10.3947/ic.2017.49.3.161 29027383 PMC5620383

[14] Martinez-Flores JA Serrano M Morales P Paz-Artal E Morales JM Serrano A. Comparison of several functional methods to evaluate the immune response on stable kidney transplant patients. J Immunol Methods. (2014) 403:62–5. 10.1016/j.jim.2013.11.019 24291342

[15] Bonora M Patergnani S Rimessi A De Marchi E Suski JM Bononi A. ATP synthesis and storage. Purinergic Signal. (2012) 8:343–57. 10.1007/s11302-012-9305-8 22528680 PMC3360099

[16] Andrikopoulou E Mather PJ. Current insights: use of immuknow in heart transplant recipients. Prog Transplant. (2014) 24:44–50. 10.7182/pit2014664 24598565

[17] Bhorade SM Janata K Vigneswaran WT Alex CG Garrity ER. Cylex ImmuKnow assay levels are lower in lung transplant recipients with infection. J Heart Lung Transplant. (2008) 27:990−4. 10.1016/j.healun.2008.06.005 18765191

[18] Pai M Denkinger CM Kik SV Rangaka MX Zwerling A Oxlade O. Gamma interferon release assays for detection of mycobacterium tuberculosis infection. Clin Microbiol Rev. (2014) 27:3–20. 10.1128/CMR.00034-13 24396134 PMC3910908

[19] Qiagen QuantiFERON®-TB Gold Plus (QFT®-Plus) ELISA Package Insert (2017).

[20] Oxford Immunotec The T-SPOT.TB Test Package Insert. Oxford Immunotec (2016).

[21] Qiagen QuantiFERON®-TB Gold (QFT®) ELISA Package Insert. (2018).

[22] Chee CBE Gan SH Khinmar KW Barkham TM Koh CK Liang S. Comparison of sensitivities of two commercial gamma interferon release assays for pulmonary tuberculosis. J Clin Microbiol. (2008) 46:1935–40. 10.1128/JCM.02403-07 18400912 PMC2446854

[23] Wigg AJ Narayana SK Anwar S Ramachandran J Muller K Chen JW. High rates of indeterminate interferon-gamma release assays for the diagnosis of latent tuberculosis infection in liver transplantation candidates. Transpl Infect Dis. (2019) 21:e13087. 10.1111/tid.13087 30927483

[24] Tagmouti S Slater M Benedetti A Kik SV Banaei N Cattamanchi A. Reproducibility of interferon gamma (IFN-γ) release assays. A systematic review. Ann Am Thorac Soc. (2014) 11:1267–76. 10.1513/AnnalsATS.201405-188OC 25188809 PMC5469356

[25] Du F Xie L Zhang Y Gao F Zhang H Chen W. Prospective comparison of QFT-GIT and T-SPOT.TB assays for diagnosis of active tuberculosis. Sci Rep. (2018) 8:5882. 10.1038/s41598-018-24285-3 29651163 PMC5897568

[26] Hornum M Mortensen KL Kamper AL Andersen AB. Limitations of the QuantiFERON-TB gold test in detecting mycobacterium tuberculosis infection in immunocompromised patients. Eur J Intern Med. (2008) 19:137–9. 10.1016/j.ejim.2007.03.020 18249311

[27] Kim TY Chang HE Lee SW Seo SH Hong YJ Park JS. A novel strategy for interpreting the T-SPOT.TB test results read by an ELISPOT plate imager. PLoS ONE. (2019) 14:e0222920. 10.1371/journal.pone.0222920 31553764 PMC6760805

[28] Schmidt T Schub D Wolf M Dirks J Ritter M Leyking S. Comparative analysis of assays for detection of cell-mediated immunity toward cytomegalovirus M. tuberculosis in samples from deceased organ donors. Am J Transplant. (2014) 14:2159–67. 10.1111/ajt.12787 25040687

[29] Walker S Fazou C Crough T Holdsworth R Kiely P Veale M. *Ex vivo* monitoring of human cytomegalovirus-specific CD8+ T-cell responses using QuantiFERON-CMV. Transpl Infect Dis. (2007) 9:165–70. 10.1111/j.1399-3062.2006.00199.x 17462006

[30] Barabas S Spindler T Kiener R Tonar C Lugner T Batzilla J. An optimized IFN-γ ELISpot assay for the sensitive and standardized monitoring of CMV protein-reactive effector cells of cell-mediated immunity. BMC Immunol. (2017) 18:195. 10.1186/s12865-017-0195-y 28270111 PMC5339961

[31] Oxford Immunotec The T-SPOT.CMV Test Package Insert. Oxford Immunotec (2017).

[32] Tanguay S Killion JJ. Direct comparison of ELISPOT and ELISA-based assays for detection of individual cytokine-secreting cells. Lymphokine Cytokine Res. (1994) 13:259–63. 7999925

[33] T-Track® CMV (2020). Available online at: https://www.lophius.com/products/t-track-cmv-elispot-kit.html (accessed August 17, 2020).

[34] QuantiFERON®-CMV ELISA Package Insert,. (2020). Available online at: https://www.quantiferon.com/wp-content/uploads/2018/10/L1075110-R05-QF-CMV-ELISA-IFU-CE.pdf (accessed August 17, 2020).

[35] Moher D Liberati A Tetzlaff J Altman DG Group TP Preferred reporting items for systematic reviews and meta-analyses: the PRISMA statement. PLoS Med. (2009) 6:e1000097 10.1371/journal.pmed.1000097 19621072 PMC2707599

[36] Liberati A Altman DG Tetzlaff J Mulrow C Gøtzsche PC Ioannidis JPA. The PRISMA statement for reporting systematic reviews and meta-analyses of studies that evaluate health care interventions: explanation and elaboration. PLoS Med. (2009) 6:e1000100. 10.1371/journal.pmed.1000100 19621070 PMC2707010

[37] Sterne JAC Hernán MA Reeves BC Savović J Berkman ND Viswanathan M. ROBINS-I: a tool for assessing risk of bias in non-randomised studies of interventions. BMJ. (2016) 355:i4919. 10.1136/bmj.i4919 27733354 PMC5062054

[38] Sterne JAC Savovic J Page MJ Elbers RG Blencowe NS Boutron I. RoB 2: a revised tool for assessing risk of bias in randomised trials. BMJ. (2019) 366:l4898. 10.1136/bmj.l4898 31462531

[39] Ravaioli M Neri F Lazzarotto T Bertuzzo VR Di Gioia P Stacchini G. Immunosuppression modifications based on an immune response assay: results of a randomized, controlled trial. Transplantation. (2015) 99:1625–32. 10.1097/TP.0000000000000650 25757214

[40] Kim SH Lee SO Park IA Kim SM Park SJ Yun SC. Isoniazid treatment to prevent TB in kidney and pancreas transplant recipients based on an interferon-gamma-releasing assay: an exploratory randomized controlled trial. J Antimicrob Chemother. (2015) 70:1567–72. 10.1093/jac/dku562 25608587

[41] Westall GP Cristiano Y Levvey BJ Whitford H Paraskeva MA Paul E. A randomized study of quantiferon cmv-directed vs. fixed-duration valganciclovir prophylaxis to reduce late CMV after lung transplantation. Transplantation. (2019) 103:1005–13. 10.1097/TP.0000000000002454 30247316

[42] Poglajen G Zemljic G Frljak S Okrajšek R Šebeštjen M Cerar A. Quantiferon-CMV guided virostatic prophylaxis after heart transplantation. J Hear Lung Transplant. (2019) 38:S119. 10.1016/j.healun.2019.01.279 32005602

[43] Kumar D Mian M Singer L Humar A. An interventional study using cell-mediated immunity to personalize therapy for cytomegalovirus infection after transplantation. Am J Transplant. (2017) 17:2468–73. 10.1111/ajt.14347 28500691

[44] Jarque M Crespo E Melilli E Gutierrez A Moreso F Guirado L. Cellular immunity to predict the risk of cytomegalovirus infection in kidney transplantation: a prospective, interventional, multicenter clinical trial. Clin Infect Dis. (2020). 10.1093/cid/ciz1209. [Epub ahead of print]. 32076718

[45] NIH The Role of ImmuKnow^®^ in the Management of Immunosuppressants in the Renal Transplant Patient. Charleston, WV: NIH (2011).

[46] Paez-Vega A Cantisan S Vaquero JM Vidal E Luque-Pineda A Lobo-Acosta MA. Efficacy and safety of the combination of reduced duration prophylaxis followed by immuno-guided prophylaxis to prevent cytomegalovirus disease in lung transplant recipients (CYTOCOR STUDY): an open-label, randomised, non-inferiority clinical trial. BMJ Open. (2019) 9:e030648. 10.1136/bmjopen-2019-030648 31420397 PMC6701703

[47] ILTS Randomized Study Comparing QuantiFERON-CMV Based vs. Standard Cytomegalovirus (CMV) Surveillance Protocol in Pre-emptive Therapy for Cytomegalovirus Prevention After Renal. (2018). Available online at: https://www.cochranelibrary.com/central/doi/10.1002/central/CN-01898092/full (accessed May 25, 2020).

[48] NIH Cell-Mediated Immunity Based Primary Prophylaxis for CMV Infection in Organ Transplant Recipients. Toronto, ON: NIH (2016). 10.1097/TP.0000000000005173 39160648

[49] NIH *Randomized Clinical Trial, Open, Multicenter Parallel, no Suspension Inferiority Prophylactic Treatment With Valganciclovir in Kidney Transplant CMV-seropositive Cellular Immunity to Develop CD8* + *CMV-Specific Treatment After Induction Thymoglobulin*. NIH (2016).

[50] NIH Cell-mediated Immunity for Prevention of CMV Disease. Lausanne: NIH (2015).

[51] van Delden C Stampf S Hirsch HH Manuel O Meylan P Cusini A. Burden and timeline of infectious diseases in the first year after solid organ transplantation in the swiss transplant cohort study. Clin Infect Dis. (2020). 10.1093/cid/ciz1113. [Epub ahead of print]. PMC7583409 31915816

[52] Neuwirt H Rudnicki M Schratzberger P Pirklbauer M Kronbichler A Mayer G Immunosuppression after renal transplantation. Memo Mag Eur Med Oncol. (2019) 12:216–21. 10.1007/s12254-019-0507-4

[53] Gardiner BJ Chow JK Brilleman SL Peleg AY Snydman DR. The impact of recurrent cytomegalovirus infection on long-term survival in solid organ transplant recipients. Transpl Infect Dis. (2019) 21:e13189. 10.1111/tid.13189 31581352

[54] Ortega F Valdés C Ortega T Quality of life after solid organ transplantation. Transplant Rev. (2007) 21:155–70. 10.1016/j.trre.2007.06.002

[55] Rodrigo E Lopez-Hoyos M Corral M Fabrega E Fernandez-Fresnedo G San Segundo D. ImmuKnow as a diagnostic tool for predicting infection and acute rejection in adult liver transplant recipients: a systematic review and meta-analysis. Liver Transplant. (2012) 18:1245–53. 10.1002/lt.23497 22740321

[56] Wang Z Liu X Lu P Han Z Tao J Wang J. Performance of the immuknow assay in differentiating infection and acute rejection after kidney transplantation: a meta-analysis. Transplant Proc. (2014) 46:3343–51. 10.1016/j.transproceed.2014.09.109 25498049

[57] Fishman JA Infection in organ transplantation. Am J Transplant. (2017) 17:856–79. 10.1111/ajt.14208 28117944

[58] Kotton CN Kumar D Caliendo AM Huprikar S Chou S Danziger-Isakov L. The third international consensus guidelines on the management of cytomegalovirus in solid-organ transplantation. Transplantation. (2018) 102:900–31. 10.1097/TP.0000000000002191 29596116

[59] Kim SH. Interferon-gamma release assay for cytomegalovirus (IGRA-CMV) for risk stratification of posttransplant cmv infection: is it time to apply IGRA-CMV in routine clinical practice? Clin Infect Dis. (2020). 10.1093/cid/ciz1211. [Epub ahead of print]. 32076699

[60] Subramanian AK Theodoropoulos NM. Mycobacterium tuberculosis infections in solid organ transplantation: Guidelines from the infectious diseases community of practice of the American Society of Transplantation. Clin Transplant. (2019) 33:e13513. 10.1111/ctr.13513 30817030

